# New Virus Genome Sequences of the Guama Serogroup (Genus *Orthobunyavirus*, Family *Bunyaviridae*), Isolated in the Brazilian Amazon Region

**DOI:** 10.1128/genomeA.01750-16

**Published:** 2017-03-02

**Authors:** Valéria L. Carvalho, Márcio R. T. Nunes, Daniele B. A. Medeiros, Sandro Patroca da Silva, Clayton P. S. Lima, Davi T. Inada, Jedson F. Cardoso, João L. S. G. Vianez, Sueli Guerreiro Rodrigues, Pedro F. C. Vasconcelos

**Affiliations:** a Department of Arbovirology and Hemorrhagic Fevers, Evandro Chagas Institute, Ministry of Health, Ananindeua, Pará, Brazil; b Center for Technological Innovation, Evandro Chagas Institute, Ministry of Health, Ananindeua, Pará, Brazil

## Abstract

This is the first announcement of two nearly complete viral genome sequences belonging to the Guama serogroup (genus *Orthobunyavirus*, family *Bunyaviridae*) isolated in the Brazilian Amazon region: *Mirim virus* (MIRV; BEAN7722) and *Ananindeua virus* (ANUV; BEAN109303).

## GENOME ANNOUNCEMENT

The family *Bunyaviridae* is composed of five genera, *Orthobunyavirus, Nairovirus, Phlebovirus*, *Hantavirus*, and *Tospovirus*. The Guama serogroup belongs to the *Orthobunyavirus* genus, composed of tripartite, single-stranded, and negative-sense RNA genomes, namely, large (L), medium (M), and small (S) genomes. The L-RNA encodes the RNA-dependent RNA polymerase. The M-RNA encodes two glycoproteins, Gn and Gc, and a nonstructural protein, NSm. The S-RNA encodes the nucleocapsid protein (N) and usually a nonstructural protein, NSs (1).

The Guama serogroup was originally described in 1961 by Whitman and Casals, and the first identified viruses were *Guama virus* (GMAV) and *Catu virus* (CATUV). Nowadays, this group of viruses has other members identified: *Mirim virus* (MIRV), *Ananindeua virus* (ANUV), *Timboteua virus* (TBTV), *Bertioga virus* (BERV), *Cananeia virus* (CNAV), *Itimirim virus* (ITIV), *Guaratuba virus* (GTBV), and *Mahogany hammock virus* (MHV). Two of these viruses, GMAV and CATUV, present relevance for public health because they cause acute febrile disease in humans characterized by fever of sudden onset, headache, arthralgia, myalgia, photophobia, asthenia, and other symptoms (1–3).

The MIRV BEAN7722 was isolated in 1957 from a sentinel monkey (*Cebus apella*) in a forest area of the Agronômico do Norte Institute (now Brazilian Agricultural Research Corporation [Embrapa]), in Belém, Pará, Brazil. The ANUV BEAN109303 was isolated in 1966 from a marsupial (*Caluromys philander*) in Brazil (Utinga Forest, Belém municipality, Pará State) (2, 4).

Brain tissues of newborn mice infected with prototype strains of MIRV and ANUV were propagated into Vero cells, and after 2 to 6 days postinfection, when the cells were showing approximately 90% cytopathic effect (CPE), the supernatant was harvested and treated with DNase to digest cellular DNAs. The RNA was extracted by MagNA Pure LC 2.0 equipment (Roche Life Science) using the MagNA Pure LC total nucleic acid isolation kit (Roche Life Science). Afterward, we followed all steps of the 454 pyrosequencing protocols (Roche): cDNA library construction, emulsion PCR-titration, emulsion PCR, and sequencing (5).

The sequences were assembled using the GS *de novo* Assembler (Newbler version 2.6), and analyses were realized using the software Geneious version 6.1.4. Here, we announced the first report of complete genome sequence (coding region) of the orthobunyaviruses MIRV and ANUV.

### Accession number(s).

This whole-genome shotgun project has been deposited in GenBank under the following accession numbers: MIRV, KY013487 to KY013489, and ANUV, KY013484 to KY013486.
